# Comparison of pre and post-operative stresses among Indian patients undergoing intra-oral grafting procedures

**DOI:** 10.6026/97320630019484

**Published:** 2023-04-30

**Authors:** Giri Senthil, Sahana Selvaganesh, Thiyaneswaran Nessapan, Vishnu Priya Veeraragavan, Rajalakshmanan Eswaramoorthy

**Affiliations:** 1 Department of Implantology , Saveetha Dental College and Hospitals, Saveetha Institute of Medical and Technical Science(SIMATS), Saveetha university, Chennai 600077, India; 2 Department of Biochemistry , Saveetha Dental College and Hospitals, Saveetha Institute of Medical and Technical Science(SIMATS), Saveetha university, Chennai 600077, India; 3 Department of Biomaterials (Green Lab), Saveetha Dental College and Hospitals, Saveetha Institute of Medical and Technical Science(SIMATS), Saveetha university, Chennai 600077, India

**Keywords:** pre and post-operative stresses, Indian patients, intra-oral grafting procedures

## Abstract

With the advent of implantology as an important means to rehabilitate the edentulous patients, the neglect over the years post extraction leads to severe bone loss. This can be corrected by grafting procedures. Grafting procedures are complex as it takes into account various factors such as the type of graft material, surgical site, surgeon's skill and the patient's compliance. Totally 20 patients ( male n=10 ; female n=10) were included in the study, 14 patients underwent GBR, 4 patients underwent direct sinus lifts and 2 underwent block grafts. The stress levels of the patient also is an important factor to consider as varying cortisol levels will be detrimental to the healing of the surgical site. Premedication with painkillers and corticosteroids can help patients comply with the treatment and feel less anxious. The stress levels of the patient were assessed using State trait anxiety index, 50.6± 6.9 was the pre-operative stress and 36.95± 9.3 was the post-operative stress. Stress levels were significantly greater before the procedure than after it, according to statistical analysis. The females were significantly having increased stress both pre and post-operatively. Also, the patients in the age group of 41-50 years showed increased amounts of stress.

## Background:

Anxiety is an emotional reaction defined as the tension, stress apprehension caused due to the fear, or any approaching danger accompanied by the activation of the autonomic nervous system [1]. Patients with anxiety are uncooperative during the procedure either implant placement or grafting procedure. The local anaesthesia for these patients are not long acting and they require more dosage compared with the patients not having anxiety or stress. Oral surgery usually generates a very high level of anxiety to patients, mainly associated with subsequent postoperative pain and morbidity [2,3]. It has been attributed to injection of local anaesthetic, use of dental hand piece and extraction of teeth. This anxiety usually impacts on the pre- and postoperative clinical healing and adherence to treatment appointments in patients who normally have to face more and longer periods of surgical appointments, in turn leading to worsening of their health indicators and the quality of life. Various definitions of anxiety have been used for different dental procedures. It can be defined as an individual and subjective experience of multi-systemic response to a threatening belief, which constitutes as a barrier to the request for oral care. In the literature, several descriptive studies have been reported with details on the importance of quantitative measurement of anxiety in different dental operative procedures using the visual analogue scale (VAS) [4] for pain and the State-Trait Anxiety Inventory (STAI) [5]. The STAI questionnaire comprises two separate scales: the anxiety-trait (STAI-T) and the anxiety-state (STAI-S). The anxiety-trait is a stable tendency of anxiety of an individual during any situation perceived as threat. The anxiety-state is temporary, arising during various subjective stresses, apprehension and hyperactivity of the autonomic nervous system, which varies in time and intensity. Even Though grafting and implant surgical procedures relatively require short periods of postoperative recovery and a low rate of complications, the physical and psychological impact on patients can make it a stressful experience. Our team has extensive knowledge and research experience that has translate into high quality publications [6, 7, 8, 9, 10, 11, 12, 13, 14, 15]. Therefore, it is of interest to assess the anxiety-trait and anxiety-state, to analyse the changes in the pre-operative and post-operative anxiety of the patients undergoing grafting procedures especially for implant placement.

## Materials and Methods:

This study is done at the Saveetha dental college, the study population was the patients who underwent grafting procedures such as guided bone regeneration (GBR), Socket preservation, and Sinus lifts procedures for implant placement after a period of 3 months from September 2020 - December 2020. The preoperative diagnosis and CBCT were taken to analyse the patient's bone three dimensionally and all the blood workup was done 1 week prior to the date of scheduled surgery. For, GBR procedure, full thickness mucoperiosteal flaps were elevated and either block graft's were harvested from the symphysis or autogenous particulate bone scrapings were obtained, for the rest particulate xenograft were used. For Socket preservation procedure, the tooth in question was extracted and the socket is examined for any granulation tissue, if present curetted out later on the socket is grafted with particulate graft and collagen membrane used for the graft stabilisation (Jason membrane).For sinus lifts, patients undergoing direct as well as indirect sinus were considered in this study. Pre-operatively patients were given the STAI questionnaire STAI-T and STAI-S were taken before the surgery on the same day as the surgery and STAI-S was repeated 3 days and 1 week after the procedure. STAI questionnaire consists of 40 questions in total and are graded according to the sum score that the patient gets. It is graded as "no or low anxiety" (20-37), "moderate anxiety" (38-44), and "high anxiety" (45-80).

[1] High level anxiety >65

[2] Moderate high anxiety 56-65

[3] Medium anxiety 46-55

[4] Minor anxiety 36-45

[5] Low level anxiety <35 

Totally 20 patients (10: male; 10: female) were included in the study, 14 patients underwent GBR, 4 patients underwent direct sinus lifts and 2 underwent block graft's. Patients were categorized based on their gender and their pre and post-operative stresses were assessed and compared.

## Results:

**Figure 1:**
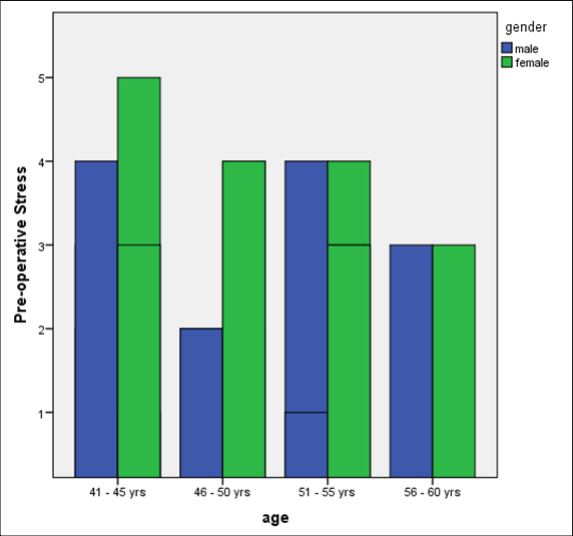
Bar graph representing the pre-operative stresses between male and female patients categorised based on their age.

**Figure 2:**
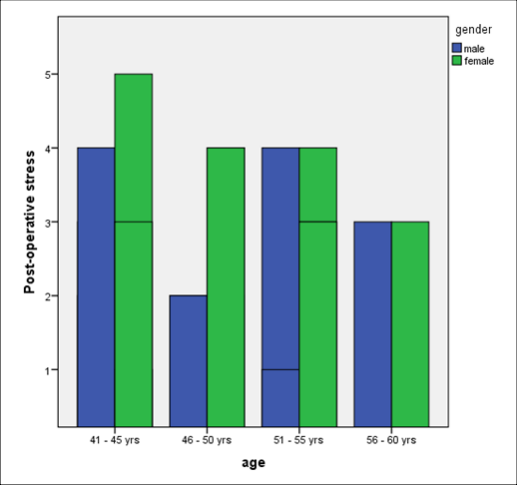
Bar graph representing the pre-operative stresses between male and female patients categorised based on their age.

The data was tabulated and SPSS software was used for the statistical analysis. Paired T-Test was used to compare the pre and post-operative stresses for all the patients and also among males and females.The mean and the SD of stress of patients, pre-operatively found to be 50.6 ± 6.9 and post-operatively 36.9±9.3 (Table 1). Pre-operative stresses were at a higher level when compared with the post-operative stresses of patients undergoing grafting procedures and the results were statistically significant (p<0.05). Also the female patients in the age group of 41-45 years had an increased amount of stress when in comparison with the male patients preoperatively (figure 1). Post-operatively the female patients showed increased anxiety in the 41-45 years age group (figure 2).

## Discussion:

Environment and family background of the patients (theory of model learning) intend to have some effect on expressing fear and the development of the same into the varying levels of dental anxiety. Another study reported that one-third of the patients introduced their fear of injection as the main reason for their anxiety. In our study also most of the patient's fears might be to some extent to the injection procedure and also due to the fear of sight of blood. A significant relation has been found between gender and stress of participants in this study. The relation of anxiety and depression with the amount of bleeding, pain intensity and difficulty in eating were not statistically significant. Many clinical studies indicate that dental implant surgeries also cause a different range of anxiety. Many studies have reported higher anxiety scores of women than men. Women easily display their feelings of fear during dental treatment better when in comparison with men. Women have more fear of surgery and dental procedures but they are more regular to dental visits and appointments. Many authors believe gender could be an influence on the capacity to manage fear. Fear is a known reason for the patients to skip the dental appointments. Huge *et al*. reported that of the many non-educated patients seeking various dental treatments, they are more prone to excessive fear. According to their findings, education had no statistically significant impact on the anxiety and stress level. If the patient is detailed about the procedure beforehand the anxiety and stress levels would not vary between the educated and uneducated sects of patients seeking dental treatment. Lindeboom and van Wijk compared flapless and flapped implant surgical techniques and the patients had more tolerable experience in flapless surgery. As this study utilizes only flapped methods for grafting, unless full thickness flaps are elevated, grafting procedure cannot be done. Fear and stress for the patients preoperatively were increased. It should be noted that current study does not represent the total population in terms of sex, age, and education distribution and is limited to a small part of a population, and hence more studies on larger samples is necessary for further evaluations.

## Conclusion:

Patients undergoing grafting procedures have an increased preoperative stress than post operatively. This can be reduced by giving patients pre-emptive analgesics and also corticosteroids which can aid in better compliance during the surgical procedure. Proper treatment planning of the case and clear explanation to the patient and reassuring the patients will also help to reduce the pre-operative anxiety.

## Figures and Tables

**Table 1:** Represents the mean and standard deviation of pre-operative and post-operative stresses.

	**N**	**Mean ± Standard deviation**	**Sig(two tail)**
Preoperative	20	50.6± 6.9	0.00*
Postoperative	20	36.95± 9.3	0.00*
*Significance <0.05
